# afspm: A framework for manufacturer-agnostic automation in scanning probe microscopy

**DOI:** 10.3762/bjnano.17.45

**Published:** 2026-05-18

**Authors:** Nicholas J Sullivan, Julio J Valdés, Kirk H Bevan, Peter Grutter

**Affiliations:** 1 Division of Materials Engineering, Faculty of Engineering, McGill University, Montreal H3A 2T8, Canada; 2 Department of Physics, McGill University, Montreal H3A 2T8, Canada; 3 National Research Council Canada, Digital Technologies Research Centre, Ottawa ON K4A 0S2, Canada

**Keywords:** atomic force microscopy, automation, manufacturer-agnostic, scanning probe microscopy, software framework

## Abstract

Scanning probe microscopy (SPM) is a valuable technique by which one can investigate the physical characteristics of the surfaces of materials. However, its throughput is hampered by the time-consuming nature of running an experiment and the significant domain knowledge required. Recent studies have shown the value of multiple forms of automation in improving this, but their use is limited due to the difficulty of integrating them with SPMs other than the one it was developed for. We have built a framework that allows for tasks written with it to be run on multiple SPM systems. Our framework defines generic commands and data structures that are passed among separate software processes, with the final SPM commands sent to the microscope after passing through an SPM-specific translator. The use of configuration files allows automation logic to be further decoupled from experiment specifics. A mediation layer permits background tasks to intermittently control the microscope, fixing detected experiment problems. Our framework has been tested on various SPM controllers, validating that it can integrate with all existing scripting interfaces types. An automated experiment was run to ensure overall running beyond integration testing.

## Introduction

In scanning probe microscopy (SPM), an atomically sharp tip is scanned above a surface of interest while measuring one or more properties. This process allows for atomic-level imaging of properties, spectroscopic analysis, and even manipulation of a sample toward atomic-scale manufacturing [1]. However, a number of factors limit higher throughput of these techniques. First, running an experiment traditionally involves constant user attention, requiring frequent monitoring and manipulation of SPM parameters. Second, limiting the decision-making of an experiment to the choices of a single researcher constrains the statistical understanding of what is being analyzed. Last, preparing, running, and analyzing such experiments requires significant domain knowledge and expertise.

Dozens of works over the past decade have investigated SPM automation. Some have shown probe tip conditioning to ensure proper surface characterization and/or manipulation [2–4]. Others have classified surface structure, detecting atoms [5], molecules [6], or defects of interest [7–8]. Even the design of experiments has been researched, using statistics to drive decisions during the experiment [9–12]. Active learning, where a machine learning algorithm’s internal model is updated during the experiment, has been used in SPM for 2D scan efficiency [10] as well as in SPM spectroscopy [11]. Furthermore, investigators have used hypothesis learning, where a model chooses between a number of hypotheses by testing them during the experiment [12]. As an example of a complete autonomous system, we highlight [13], where atomic-level characterization is achieved via, among other processes, a thermal drift correction approach first described in [14].

Despite promising results, a lingering issue common to all these techniques is reusability. In most cases, the source code is written for a specific SPM controller’s commands and data structures; adapting the code to a different controller is a time-consuming and non-trivial task. Some work has been done in this regard: In [15], a software ecosystem was developed to simplify reading and analyzing data from various scientific instruments. While a generalized approach to data handling solves part of the problem, it does nothing to address the control needs posed across the various SPM controllers. Another contribution was made in [16], where a hardware–software framework enables automated experimentation. This framework focuses on the design of low-level particulars, to give researchers finer control on aspects of an experiment (e.g., scanning trajectories and excitation waveforms); while useful, it requires a separate hardware component. These developments complement ours.

Outside of nanoscience, the particle accelerator community have developed Bluesky [17], a suite of Python packages centered around defining and running multiple instrument experiments in a hardware-abstracted manner, while maintaining format-independent data and metadata storage. Outside of many-instrument orchestration, this suite provides various abstractions that may be suitable to other scientific fields. Bluesky does not solve the reusability issues described above, but may prove suitable as an interface to coordinate an SPM among other instruments.

We have developed a software framework for SPM to write reusable and shareable automation scripts, named afspm (after Automation Framework for SPM). This framework serves as a manufacturer-agnostic foundation with which automation processes can be developed; for example, the system developed in [13], a specific application demonstrated on a single SPM, can – once implemented in this framework – be more easily utilized on other SPMs. In what follows, we present an overview of the framework, showing how reusability is achieved by decoupling the various script specificities from the overarching algorithm. Afterward, we validate that our approach should support any microscope controller by integrating and testing translators for each possible scripting interface type. We show the framework functioning beyond integration testing by running an automated experiment; specifically, we implement a drift estimation approach similar to that in [14]. In doing so, we enable easier reuse of this specific application (drift correction) on other SPMs. We conclude by highlighting the value of some of our design decisions for other experimental fields.

## Framework Design Overview

**Figure 1:**
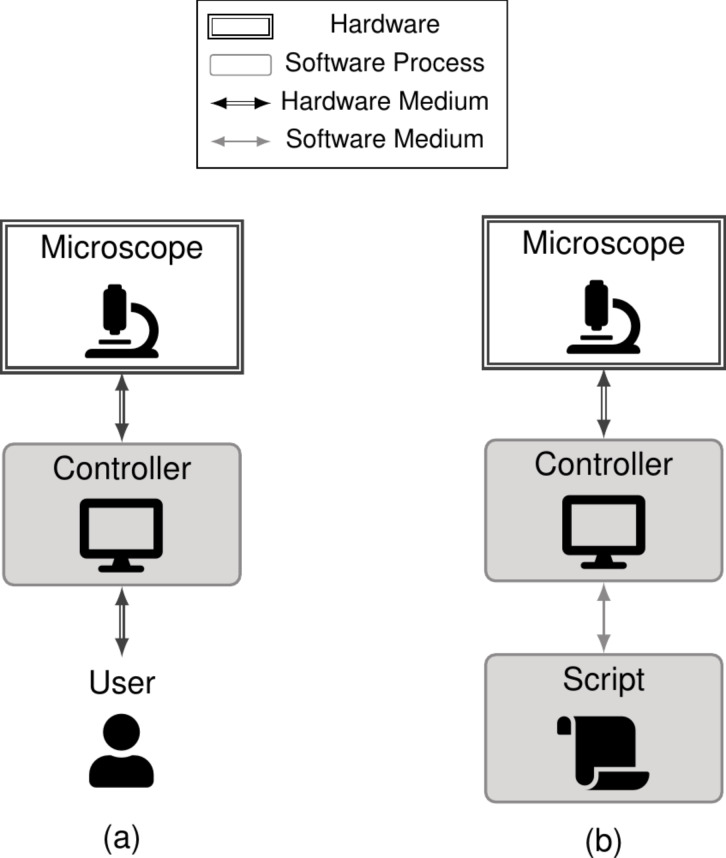
(a) Script-free experiment running, where a user interacts with the SPM controller directly, sending requests to the microscope via the microscope controller user interface. (b) Script-based experiment running, where a script replaces manual requests with programmatic ones.

Scripting aims to automate some of the user’s decisions during an experiment. As illustrated in Figure 1, this level of automation tries to replace a user’s manual interaction (Figure 1a) with equivalent programmatic commands sent to a microscope controller (Figure 1b). Note that here we refer to the software controller when we discuss the microscope controller; this software application is responsible for managing the programmatic instructions (e.g., start a scan) that are sent to the hardware controller, which handles real-time control of the microscope (e.g., sending the raster-scanning and *z*-height signals).

**Figure 2:**
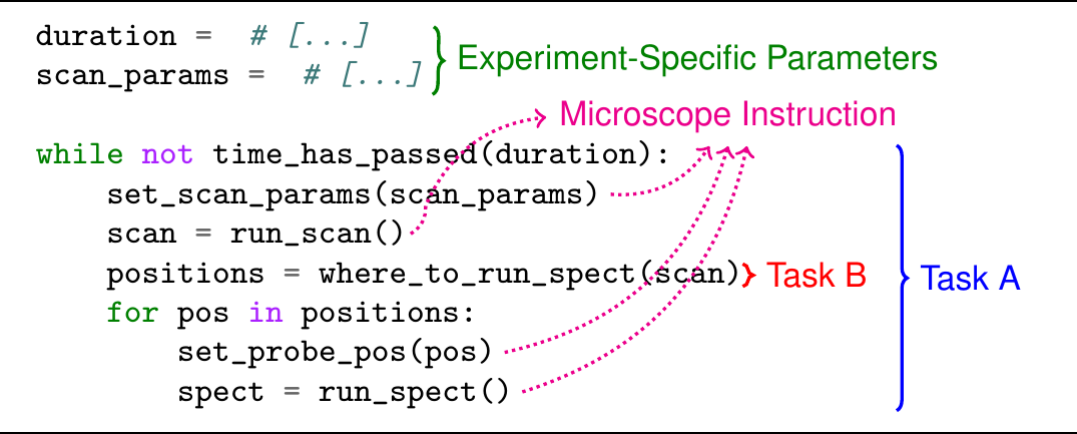
Sample script delineating various aspects of a script. Here, we see that for the set of instructions we have experiment-specific parameters, which are likely to vary from one experiment to another, and instructions that are aimed at the microscope. Additionally, a script may comprise multiple tasks.

**Figure 3:**
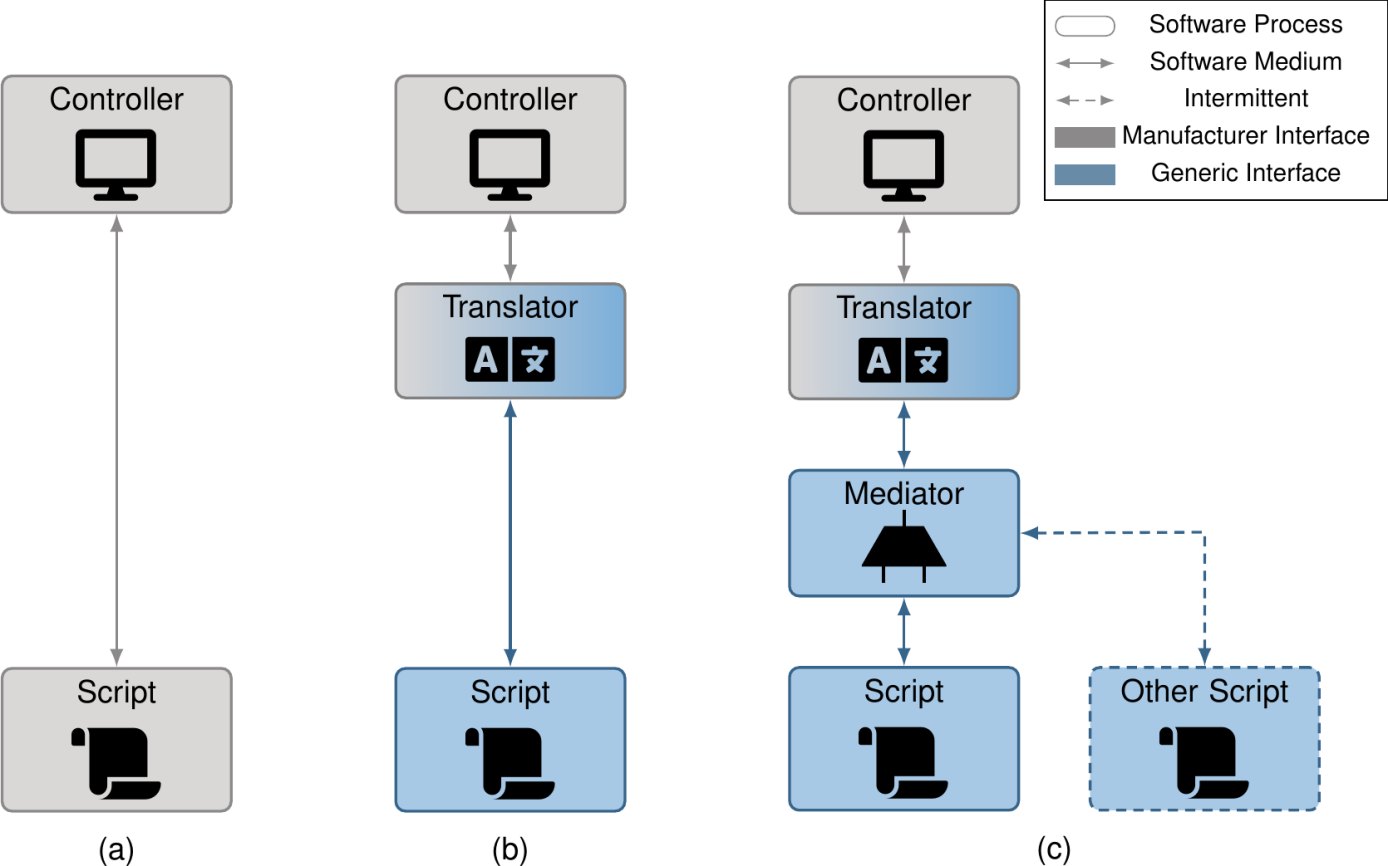
(a) Today, SPM scripts are usually written using manufacturer-specific software interfaces, requiring a complete rewrite to port to another system. (b) Usage of a similar script in afspm involves sending generic commands to a microscope translator, which translates these to manufacturer-specific commands. (c) For more involved experiments, a microscope mediator manages control to the microscope, allowing monitoring processes to intermittently take control and correct detected experiment problems. The main experiment script can then be written focused solely on the main experimental loop.

A script is an algorithm, or set of specific instructions, for performing some task (our definition here is broad, disregarding the usage of a typeless, interpreted language [18]). We delineate the various aspects of a script with an exemplary one (Figure 2), where we regularly scan a fixed region of a sample and then run spectroscopies on a set of positions we have deemed interesting from analyzing said scan. A subset of a script’s instructions are direct instructions to be sent to the microscope (delineated “Microscope Instruction” in Figure 2); these are manufacturer-specific. Another subset of the instructions have predefined parameters associated to them (in this example, “time_has_passed” and “set_scan_params”) that are experiment-specific as they are sample- or system-dependent. Last, a single script is often written for performing a composite task, that is, one comprising multiple interdependent subtasks. In this example, we have two tasks, namely, one performing the experimental loop (Task A) and one determining where to run spectroscopies based on the latest scan (Task B). Unless tasks are well decoupled, bundling them makes running them independently difficult. In all three cases, reuse becomes more difficult due their intermixing; any approach toward higher reusability would minimize this. Our approach was to develop a software framework, that is, a reusable architecture providing pre-built functionality for writing scripts [19]. Figure 3 visualizes some of its features.

As seen in Figure 3a, scripts using manufacturer-specific instructions restrict themselves to said manufacturer; reuse requires a rewrite. Our framework decouples the manufacturer-specific instructions sent in a traditional script by defining a subset of generic commands and data structures, which are translated to those of a given microscope controller via a translator (Figure 3b). Experiment-specific parameters are decoupled from algorithmic logic via the usage of configuration files: the algorithmic logic resides in scripts, while the predefined parameters reside in a configuration file. The configuration file also defines the various scripts to be instantiated for a given experiment and the mechanism by which they communicate with each other. For experiments where multiple scripts may need to communicate with the microscope, a mediation layer has been developed (Figure 3c), which provides a mechanism (declaring experiment problems) by which a secondary script can temporarily take control of the microscope. This permits background tasks to monitor the experiment and intermittently take over to fix detected problems, decoupling the main experiment from various background tasks.

Our software framework has been developed in Python [20], giving users access to the existing Python ecosystem for scientific computing. While we have defined a subset of microscope commands and data structures, the framework can be adapted as needed. Furthermore, the mechanism by which scripts communicate allows individual scripts to be written in other languages. More information can be found in sections “Communication Protocol” and “Implementing Components in Other Languages” of Supporting Information File 1.

### Decoupling manufacturer specifics

**Figure 4:**
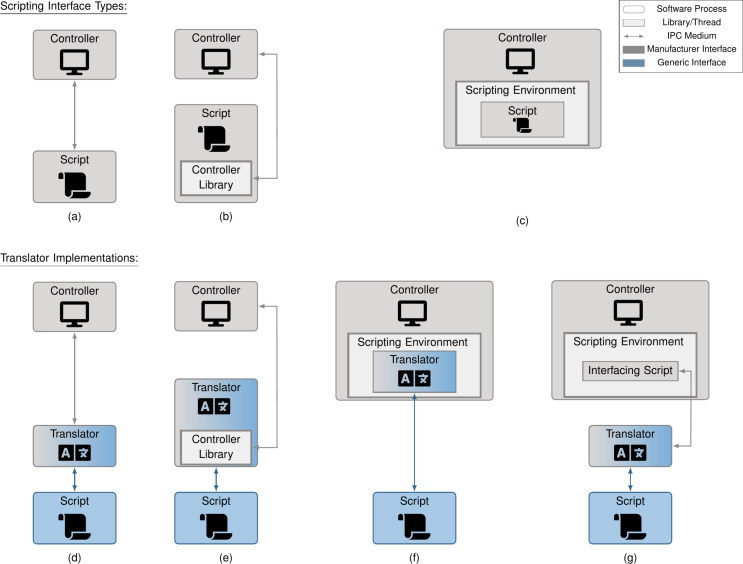
System diagrams of the various scripting interfaces types (a–c) and how microscope translators can be created for them (d–g). For IPC interfaces (a), translators can easily reuse this interface to communicate with the controller (d). For external library interfaces (b), the translator interfaces via the library (e). For internal interfaces (c), the translator may either: (f) reside within the scripting environment or (g) interface with a specific script that resides within the scripting environment. The choice of which to use is driven primarily by ease of integration. In all cases, the translator receives microscope instructions via an IPC interface receiving generic commands and data structures. Note the shift from “Software Medium” to “IPC Medium” in the legend, as we differentiate between inter-process and in-process interfaces.

In practice, controller interfaces differ not just in the commands and data structures used, but also in the programming language supported and the interfacing mechanism itself. In this section, we classify the various interfacing mechanisms and show how one may implement translators for each. As we will see, the proposed solutions allow instruction set and programming language differences to be circumvented. The various scripting mechanisms are visualized in Figure 4a–c along with translator implementations in Figure 4d–g. In Table 1, we list a sampling of controllers from various manufacturers and the scripting interfaces they expose.

**Table 1:** Listing of the programming interfaces of a selection of SPM controllers from various manufacturers. If “all” is indicated, all hardware controllers by this manufacturer should support this interface. All information was obtained via manufacturer datasheets or marketing documents and is therefore not guaranteed to be accurate.

Manufacturer	Supported Controller	Interface type	Language(s)

Asylum Research^a^	all	internal	IGOR
Bruker Nano	Nanoscope	internal	“Recipes”^b^
		external library	C
		external IPC	N/A
Createc	SC6711	external IPC	N/A
EPFL LBNI AFM	N/A	internal	LabVIEW^c^
GXSM	all	internal	Python
SPECS Nanonis^d^	Mimea BP5e/Tramea Base	internal	LabVIEW
		external IPC	N/A
Nanosurf	all	internal	VBScript
	all	external IPC	N/A
	all	external library	Python
Park Systems	SmartScan	internal	Javascript
RHK Tech	R9+	External IPC	N/A
Scienta Omicron	SXM	internal	PASCAL-like
		external library	Python
		external IPC	N/A
	MATRIX	internal	MATE
Unisoku	SPC-STG	internal	LabVIEW

^a^Oxford Instruments Asylum Research Inc., referred to as Asylum Research for convenience. ^b^A custom visual programming language for defining internal scripts. ^c^All LabVIEW Virtual Instruments (VIs) expose front panel attributes via an External IPC interface. We do not explicit this for these instruments as it is not guaranteed that all desired settings are part of the front panel. ^d^SPECS Surface Nano Analysis GmbH Nanonis, referred to as SPECS Nanonis for convenience.

With inter-process communication (IPC) interfaces (Figure 4a), scripts are run as separate processes from the microscope controller. An IPC interface allows different processes on the computer to communicate with each other; they differ from in-process communication in their generality (being language-agnostic) and their added timing delays (which could be limiting in high-frequency applications). Because this complicates scripts, some manufacturers expose a language-restricted controller library, that is, an external library interface (Figure 4b), which makes communication with the microscope easier, but restricts the script to a single, or subset of, programming languages. Other manufacturers expose an internal interface (Figure 4c), where a provided script is run within the controller process; here, a single programming language is supported and timing delays are minimized due to in-process communication.

Both the internal and external library interfaces impose programming language constraints. From Table 1, there is no common programming language among our sample of manufacturers; a change in programming language is thus required for at least some systems. This cross-language communication can be achieved via an IPC interface with each language in its own process.

Our implementation treats the script and the translator as independent processes communicating over an IPC interface via generic commands and data structures. This is most easily implemented for the IPC interface type (Figure 4d), where the script sends generic instructions over IPC to the translator, which in turn sends the translated manufacturer-specific instructions over IPC to the controller. In the case of external library interfaces, the controller library resides in the translator process (Figure 4e), with the script-translator communication involving generic instructions over IPC and the translator-controller communication occurring via the library. Last, internal library interfaces can be implemented in one of two ways: the translator logic may reside directly in the scripting environment (Figure 4f), or it may reside in a separate process (Figure 4g). In the former case, the translator-controller communication is embedded in the scripting environment. In the latter case, the translator-controller communication involves manufacturer-specific instructions over IPC, where an interfacing script is run in the scripting environment. By following the translator implementations of Figure 4d–g and utilizing generic commands and data structures, we can decouple manufacturer specifics from our scripts for any scripting interface type.

### Experiment configuration

**Figure 5:**
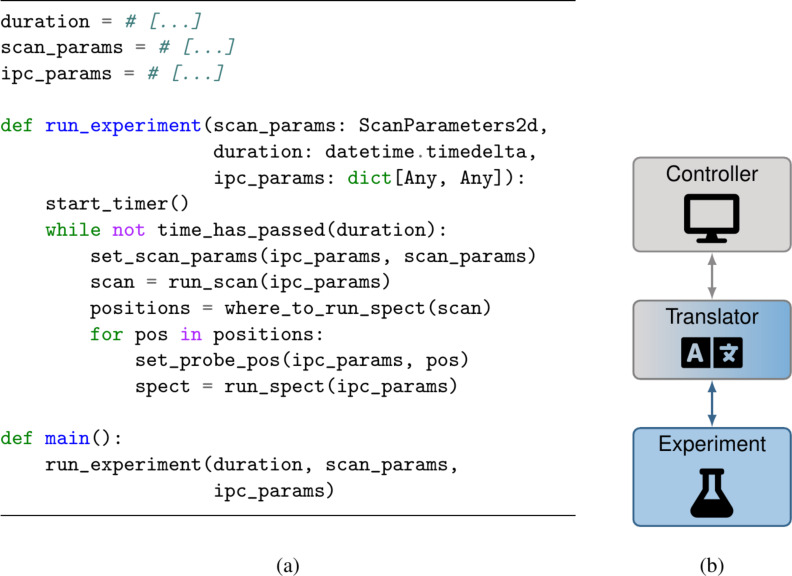
Pseudocode (a) and system diagram (b) for an experiment script talking to a translator without a configuration file. Here, experiment-specific parameters (e.g., the scan parameters), as well as communication parameters (e.g., how we talk to the translator) are included in the single script file. To run the same experiment with different experimental setup, one would modify the source file itself.

By using configuration files, afspm allows algorithmic logic to be separated from experiment-specific parameters and split up into subtasks as needed. The potential benefits are best demonstrated via variations of our earlier sample script. In Figure 5, we see pseudocode (Figure 5a) and a system diagram (Figure 5b) for a script including experiment-specific parameters within it. In coupling the algorithmic logic and experiment-specific parameters, we force a new copy of the base script for each new experiment. Imagine running the experiment with the same system and sample twice: The only likely change is the region of the sample we wish to scan (“scan_params” here), due to particulars of sample placement during setup. Even in this case, we would need to duplicate (or modify) our script, indicating the parameter change. Any bug fixes or improvements to our base script would need to be tracked among the various copies we maintained – a time-consuming and error-prone process.

**Figure 6:**
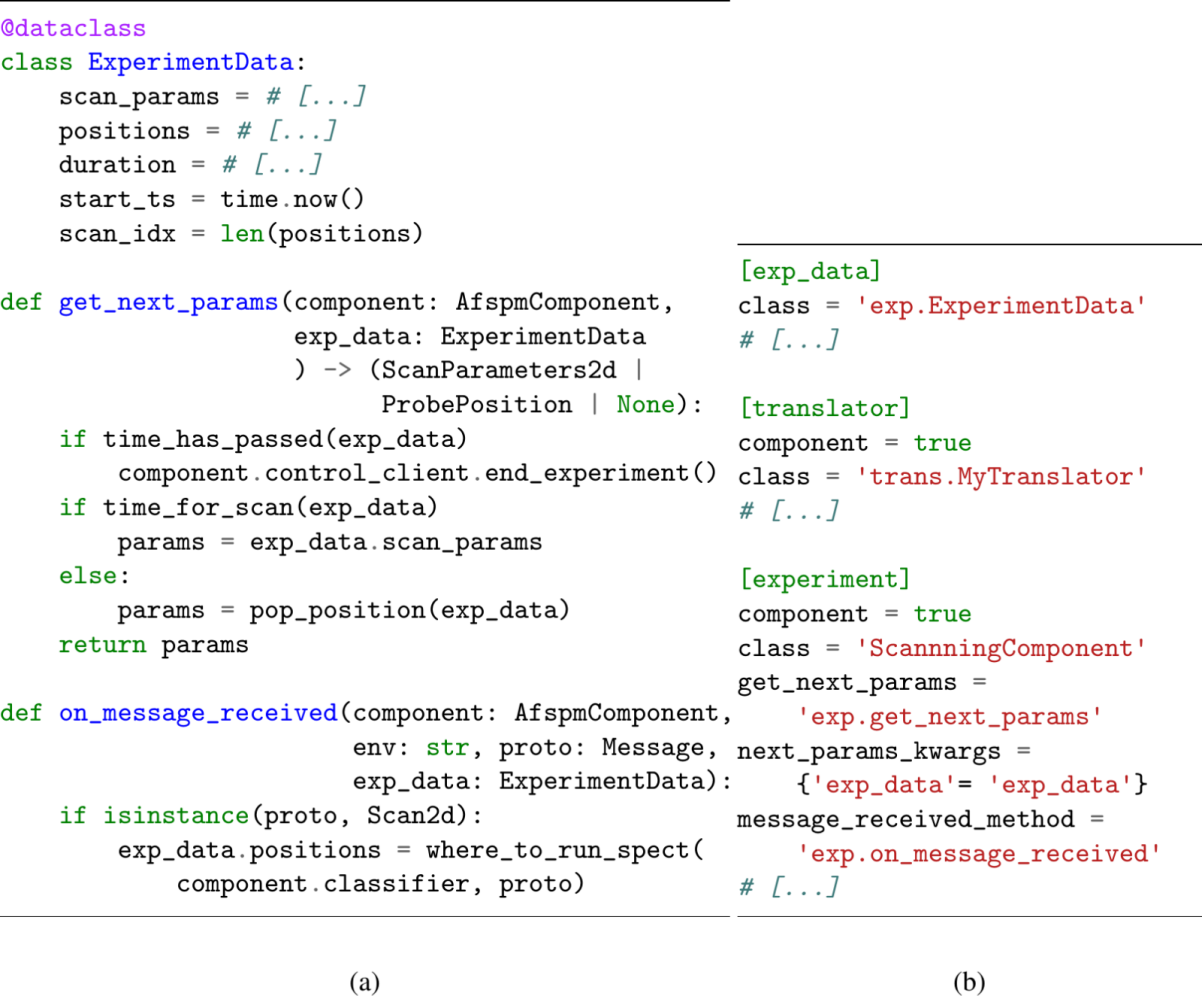
Pseudocode (a) and configuration file (b) for an experiment with system diagram matching Figure 5 using a configuration file. In the source file (a), a data structure holding experiment-specific parameters is defined (“ExperimentData”), and the logic of what action to take once a prior action has finished is described (“get_next_params”). The logic for determining where to run spectroscopies is run whenever a new scan is received (“on_message_received”). In the configuration file (b), the experimental parameters for a specific experiment and the two software processes to be instantiated are defined (“[translator]” and “[experiment]”). Note the usage of a variable “component” to indicate to the parser that this is a script to be instantiated. Additionally, how the two scripts communicate is defined (not shown here).

We can avoid this by defining the type of parameters our script expects in the script itself, and declaring them per-experiment in a separate configuration file; the script loads the parameters on startup by parsing the configuration file. This is the approach taken in afspm, as exemplified in Figure 6. In the pseudocode (Figure 6a), we see that the expected parameter types are defined in a data structure (here, “ExperimentData”), which is used by the “get_next_params” method to determine the next action to perform (scanning or running spectroscopies). The method “on_message_received” is run whenever a message is received by the script; in this case, it is used to determine where to run spectroscopies by analyzing each received scan (“Scan2d”). Both of these methods are method callbacks defined within the software framework and are called when certain events occur (i.e., event-driven logic). From the configuration file (Figure 6b), we see that in addition to defining experiment-specific parameters (“[exp_data]”), the various scripts to run are also explicited (“[translator]” and “[experiment]”). When a configuration file is loaded on startup, afspm will instantiate a new process for each indicated script and set up their communication mechanisms as defined by the configuration. Note that the communication mechanisms are hidden in this example; see section “Communication Protocol” of Supporting Information File 1 for more information.

**Figure 7:**
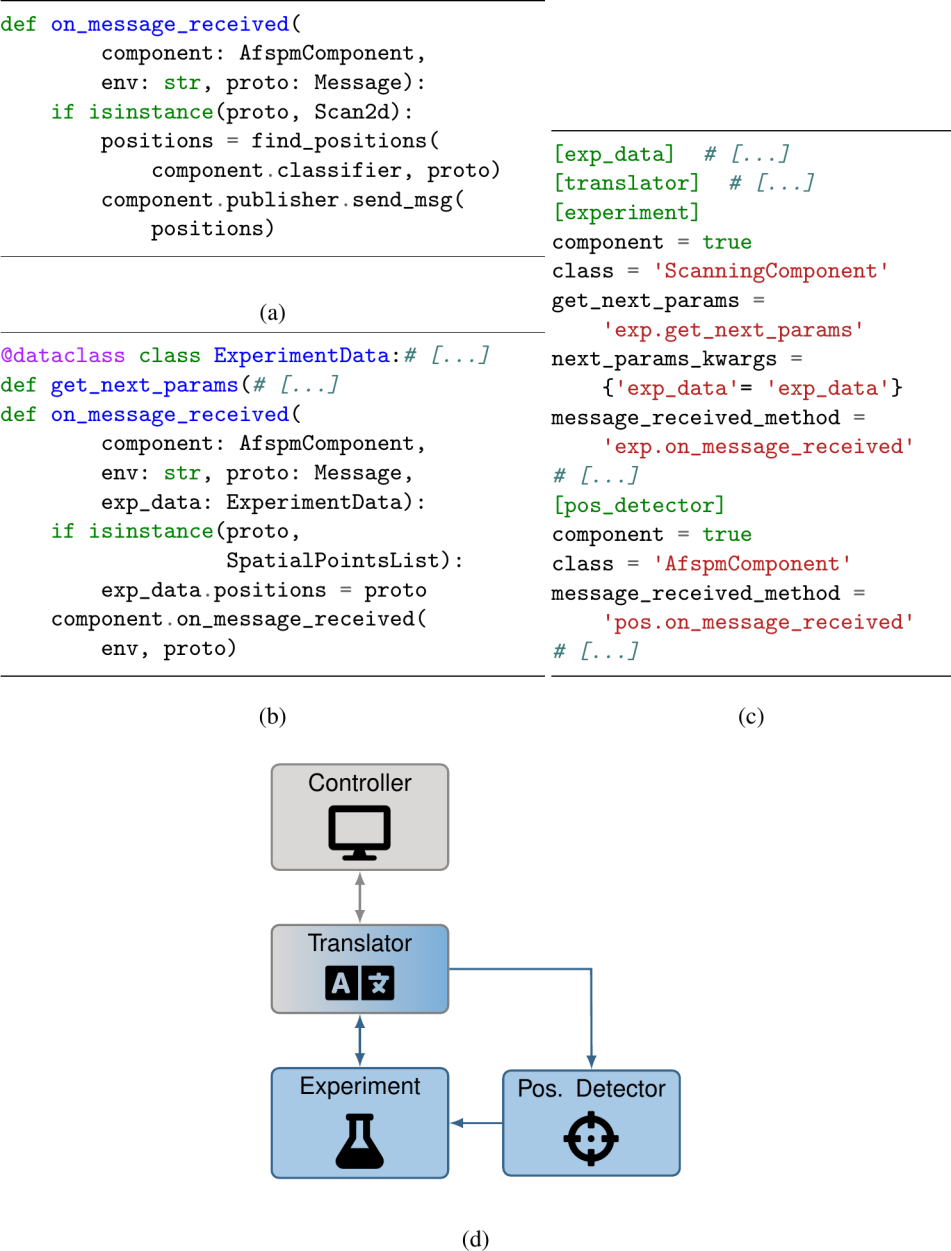
Pseudocode (a, b), configuration file (c), and system diagram (d) for an experiment involving two dependent scripts. A classifier-based position detector (a) receives scans and detects positions to perform spectroscopies. These are sent to the experiment script (b), which sends scan and spectroscopy requests to the microscope. Note that this script contains equivalent logic to that of Figure 6a, with changed logic to handle the received spectroscopy positions. The configuration file (c) differs from Figure 6c in the definition of the position detector and its input parameters.

The use of a configuration file also simplifies separating a composite task into subtasks, as seen in Figure 7. This example performs the same logic as that shown in Figure 6, but with the logic split in two tasks, that is, determination of where to run spectroscopies (Figure 7a) and general experimental logic (Figure 7b). The position detector script (“[pos_detector]” in Figure 7c) concerns itself only with analyzing received 2D scans to decide where spectroscopies should be run. One could imagine the method “find_positions” invoking a pre-trained machine learning classifier, or predefined image processing logic. The determined positions are sent over IPC to the experiment script (“[experiment]” in Figure 7c), which, upon receipt, stores them in an internal data structure for later use. This script runs the overarching experimental logic, namely, alternating between 2D scans and spectroscopies for a predetermined duration.

### Mediation logic

Thus far, we have considered experiments where only one script communicates with the microscope. However, one can envision experiments where multiple separate tasks may require microscope control. This is notably the case for tasks that involve monitoring and fixing issues detected during an experiment: Correcting for thermal drift, characterizing the tip, and optimization feedback parameters all fit within this category. To simplify automation of this kind, we have added mediation logic determining control of the microscope, managed by a microscope mediator that sits between the translator and all other running scripts (see Figure 3c). This mediation assumes a “primary” script that is principally communicating with the microscope and various “background” scripts that monitor and fix issues in an intermittent fashion.

Tied to mediation is the concept of experiment problems, predefined issues that may be flagged or removed throughout an experiment. The mediator maintains a list of currently logged experiment problems and a record of the current script in control along with the enumerated problem it resolves (an enumeration exists for “standard” scripts that do not resolve a problem). Control can only be given when it has not been granted and is determined in a greedy fashion, that is, to the first “suitable” script requesting control, either one that resolves a flagged problem if problems have been flagged or a standard script otherwise.

**Figure 8:**
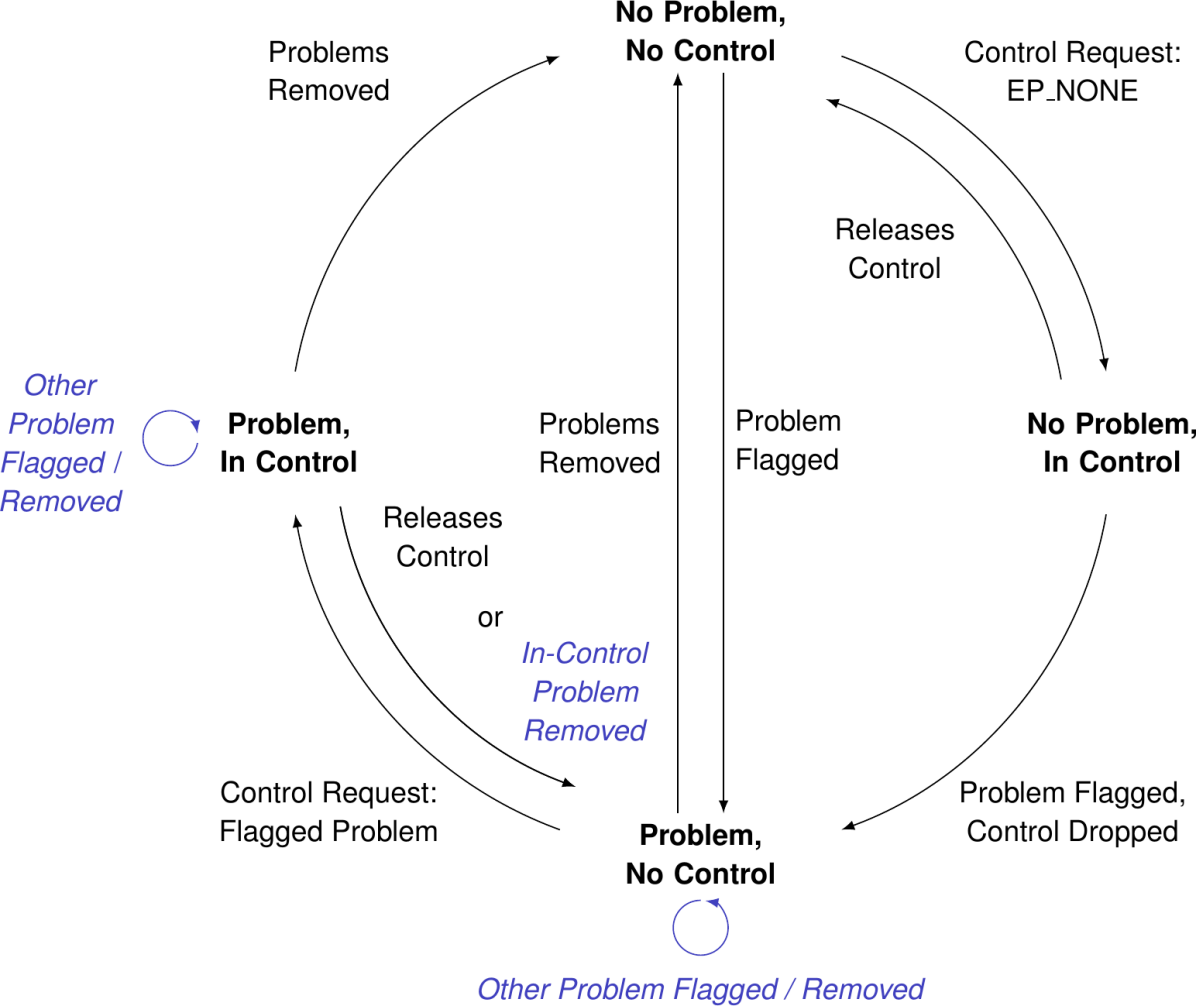
Mediation logic state machine. Here, we transition from the default “No Problem, No Control” state to “No Problem, In Control” when a script solving no specific problem requests control. If a problem is flagged, control is dropped and we transition to “Problem, No Control”. A script able to resolve the flagged problem can gain control, transitioning to “Problem, In Control”, and we return to the default state when the problem has been removed.

The mediation logic is best demonstrated via the mediator’s state machine (Figure 8), starting from top and following clock-wise. We begin with no problems flagged and no script in control (“No Problem, No Control”). At this point, a standard script can request and gain control of the microscope (“No Problem, In Control”). If there are no other scripts monitoring for experiment problems, the whole experiment may reside in this state. If a monitoring script exists and a problem is detected, it may flag it to the mediator, causing the in-control script to lose control (“Problem, No Control”). Any number of further problems may be flagged or removed in this state, as long as a single problem remains flagged. At this point, a script capable of fixing a flagged problem can request control (“Problem, In Control”). Following this, the in-control problem can be removed (or the in-control script releases control), transitioning back to “Problem, No Control”; or all flagged problems can be removed, causing the mediator to remove control and transition to “No Problem, No Control”. As indicated by the vertical transitions, any script may flag a problem and transition the mediator from “No Problem, No Control” to “Problem, No Control”; transitioning back requires all flagged problems to be removed.

In the current implementation, there is no hierarchy between experiment problems. Race conditions may thus occur whenever two suitable scripts request control. Given our assumption of a single “primary” script, this is mainly problematic if certain experiment problems are interdependent and the resolution order is important. This can be avoided by extending the mediator to accept a problem hierarchy on startup; subsequent versions of the framework may support this.

### Base microscope actions and parameters

When defining this framework, we attempted to define some minimum set of commands and data structures that we believe form a “base” set to run automation tasks. Such a definition is arbitrary; however, the high volume of controls available in most SPM controllers make the task of creating a fully comprehensive framework impractical. We chose to focus on the most common actions and parameters (Table 2); these are running and configuring scans (the physical region and data resolution), running and configuring spectroscopies (the physical position where it is to be run), and configuring the *z*-height feedback (the proportional and integral gain parameters and setpoint value).

**Table 2:** Table listing generic requests expected from all translators.

Grouping	Request	Notes

actions	start/stop 2D scan	begin or override currently configured scan
	start/stop 1D spectroscopy	begin or override currently configured spectroscopy
parameters	set scan parameters	spatial rectangle and digital resolution
	set probe position	*xy* position for 1D spectroscopy
	set *z*-control parameters	proportional / integral gain weights

Table 3 lists the generic data structures all microscope translators are expected to publish to connected processes and their source (i.e., where a translator obtains them). Here, the scan parameters, probe position, and *z*-control parameters match what is settable and described above. The scope state indicates the current state the microscope is in (e.g., scanning, moving, or free). The 2D scans structure holds the one or more 2D scans collected during a single scanning event, where each scan corresponds to a channel saved. The 1D spectroscopy structure holds a one-dimensional array of values collected at a single *xy* position within the scan region. Both the scan and spectroscopy data structures are expected to be read directly from the computer hard drive, which simplifies the translator logic as it does not need to monitor data live (which is accessible for only some controllers).

**Table 3:** Table listing generic data structures expected from all translators and where a translator may source each from.

Data structure	Source

1D spectroscopy	saved files
2D scans	saved files
probe position	microscope controller
scan parameters	microscope controller
scope state	microscope controller
*z*-control parameters	microscope controller

The exposed level of control assumes the user has pre-configured their “operating mode” (e.g., amplitude-modulated atomic force microscopy (AM-AFM) or frequency-modulated atomic force microscopy (FM-AFM)) and spectroscopic mode (e.g., *IV* curves or force spectroscopy), which we believe is justifiable as part of the experimental setup. This base set of operations gives the user a reasonable degree of flexibility: One could design an image analysis script to analyze 2D scans for defects of interest; an experiment could then regularly alternate between performing 2D scans and running local spectroscopies on the detected defects. A user of the framework is not restricted to this subset of controls; generic actions and generic parameters can be added as needed (e.g., adding actions to swap between operating modes or adding parameters to control additional feedback loops).

## Framework Validation Methodology

To validate interfacing feasibility, we developed a translator for each solution illustrated in Figure 4. In order to ensure a given translator behaves as the framework expects, an automated test suite was created that validates each of the base parameters and actions, ensuring that scripts written in our generic fashion can be run on a specific microscope. It can be easily invoked via command-line, allowing a user to run an automated sanity check before beginning an experiment. As this test suite validates correct interaction between controller and translator, the actual data recorded during a test is unimportant. We therefore consider it a best practice to run these with the tip disengaged, to avoid unnecessary risk to tip or sample.

The translator test suite exists alongside a comprehensive test suite that validates the framework and available scripts for expected functionality. At any point while the framework is installed, its general functioning, and that of all existing scripts, can be validated quickly. By providing both of these, we allow for quick automated testing, paving the way for continuous integration practices to be run on all supported microscopes. If widely adopted, nightly tests could be run to ensure all integrated microscopes remain supported as the framework evolves.

Next, we aimed to validate full functioning of the framework by developing and running an automated experiment. We chose to run a drift correction system under conditions where correction was merited. The particular drift estimation method chosen is a feature tracking one similar to that reported in [14] (we direct readers here for estimation specifics). Further information on our implementation may be found in section “Drift Correction” of Supporting Information File 1.

## Results and Discussion

### System interfacing results

**Figure 9:**
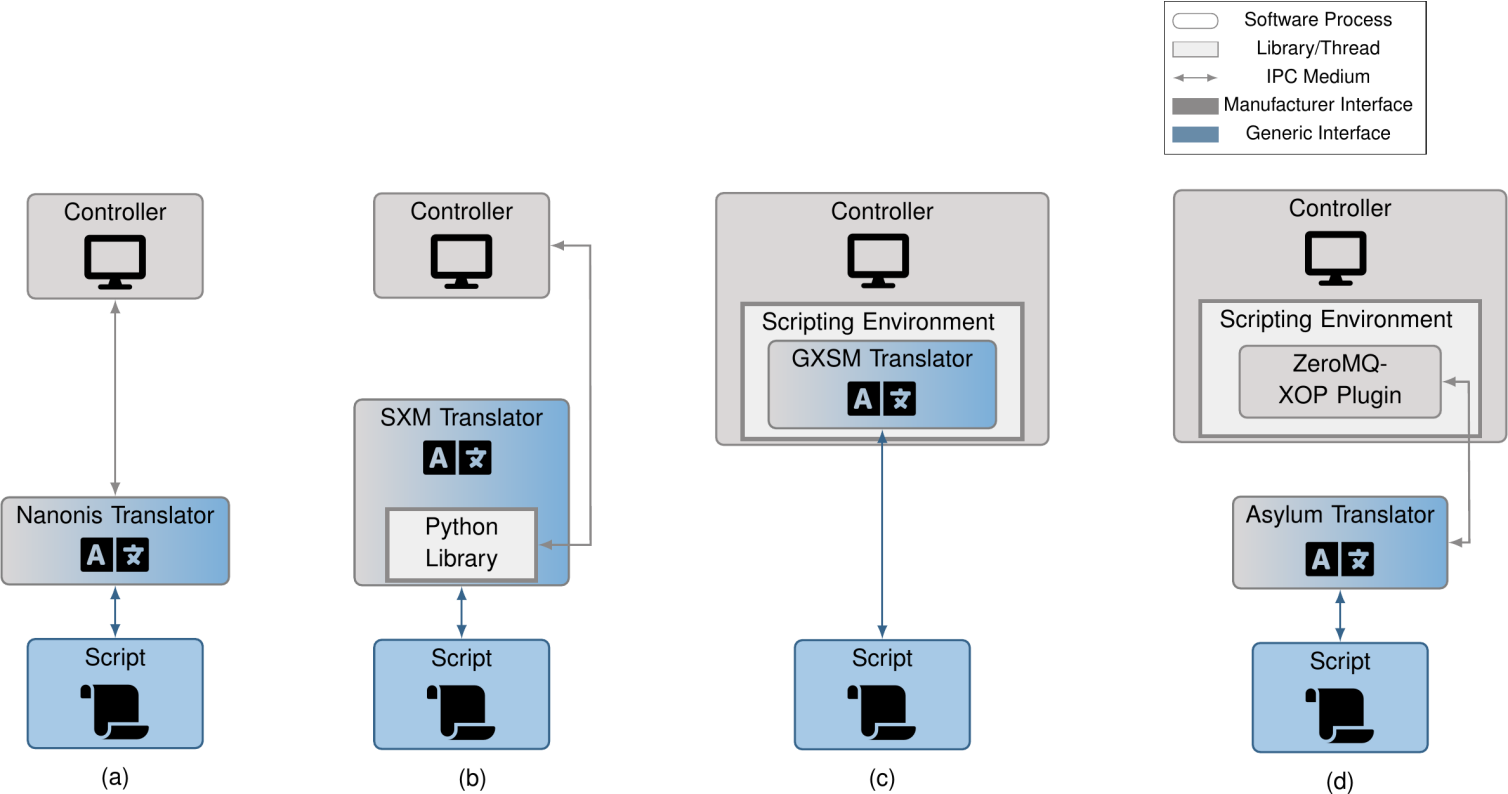
System diagrams of the translators for various integtrated microscope controllers: (a) SPECS Nanonis, (b) Scienta Omicron SXM, (c) GXSM, and (d) Oxford Instruments Asylum Research.

We developed translators for a SPECS Nanonis controller, a system using a Scienta Omicron SXM controller, one using the Gnome X Scanning Microscopy project (GXSM, an open-source software and hardware controller system), and an Asylum Research system. Using the translator test suite, we were able to validate that all translators supported all of our base microscope operations (with the temporary exception of the Scienta Omicron system, as described below). Videos showing these in action can be found in Supporting Information File 2. System diagrams of the implemented translators may be seen in Figure 9.

#### SPECS Nanonis Integration

We interfaced with a SPECS Nanonis STM Simulator, simulating a Nanonis Mimea BP5 SPM system. This software simulator mimics the hardware and software controller in a one-to-one fashion at the interface-level; to a connected translator, it is equivalent to a real system. The associated software controller provides a LabVIEW-specific library interface, and a transmission control protocol (TCP)-based IPC interface. Our translator connects via the IPC interface, as seen in Figure 9d and matching the design in Figure 4d. It translates generic commands and data structures into TCP messages as defined in the Nanonis specification, and unpacks the received responses according to said specification. For reading SPECS Nanonis scans and spectroscopies, the Python package SciFiReaders was used [21].

#### Scienta Omicron SXM Integration

Our Scienta Omicron system was the Infinity UHV SPM Lab using an SXM controller. The SXM controller provides an internal Pascal-like scripting language interface, a dynamic data exchange (DDE) IPC interface, and a series of Python scripts functioning as an external library interface. We chose to have our translator interface via the external library interface, as seen in Figure 9c and matching the design in Figure 4e. Our current implementation interfaces with an earlier version of the programming interface and software controller (version 28.8). This version is more limited in the available controls and notably does not allow for stopping scans or spectroscopies. Because of this, our implementation does not pass two of the tests in our translator test suite. We note that this is a versioning limitation and unrelated to the feasibility of interfacing with an external library interface type. A future version of our translator will interface with a later software controller version, enabling all controls validated by the test suite.

We made modifications to the provided Python script to function optimally for our translator. This modified script is included in our software framework with permission from the manufacturer. For reading SXM scans and spectroscopies, custom Python readers were written.

#### GXSM Integration

For our GXSM-controlled system, our hardware controller was a soft dB Signal Ranger MK2-810, connected to a custom non-commercial SPM system. The GXSM controller provides a Python-based internal scripting interface. In order to integrate with our framework, we designed the translator to run within the scripting environment, communicating with other scripts via our IPC interface (see Figure 9a, matching the design of Figure 4f). Minor changes were made to GXSM’s Python programming interface to simplify cancelling and restarting scripts, pushed to GXSM’s main development branch. Since a pre-existing Python package for reading GXSM scans and spectroscopies did not exist, one was written called gxsmread [22].

#### Asylum Research Integration

Our Asylum Research system was an MFP-3D-BIO, which provides an IGOR-based internal scripting interface. Due to the non-Python scripting language, we chose to use a third-party tool, “ZeroMQ-XOP” [23], to function as the interfacing script and allow for communication with the controller (see Figure 9b, matching the design in Figure 4g). This tool installs a custom plugin in the Asylum Research computer software, allowing it to receive IGOR commands packaged into a JSON message format and passed over an IPC interface. Our Asylum Translator thus translates our generic commands and data structures into IGOR commands as represented by this JSON message format, which are sent to the custom plugin using the IPC interface of “ZeroMQ-XOP”, where they are parsed and called directly inside the scripting environment.

We made minor modifications to this tool in order to support the underlying data analysis software used by Asylum Research, an earlier version of Igor PRO. These changes were pushed to “ZeroMQ-XOP”’s main development branch. For reading Asylum Research scans and spectroscopies, the Python package SciFiReaders was used [21].

### Running an automated experiment

In order to validate the functioning of our drift correction logic, we ran experiments with it on the Asylum Research MFP-3D-BIO AFM system, scanning a CD stamper as a test sample using a MikroMasch HQ:NSC15/Al AFM probe (40 N/m force constant, 325 kHz resonant frequency). These experiments were run under ambient conditions in an AM-AFM mode. Prior to engaging with the sample, we performed an auto-tuning operation to optimize the cantilever frequency and manually optimized the *z*-height feedback parameters for reasonable imaging quality. In the experiment, we attempted to scan the same region of our sample over a long period of time (∼16 h). For our drift estimation, we chose to track a channel representative of the topographic variation on the sample. For this operating mode and microscope setup, this corresponded to the “Height Retrace” channel. We used the instrument’s internal “Flatten1” scan pre-processing step, which fits an average line to each scanline and removes it.

**Figure 10:**
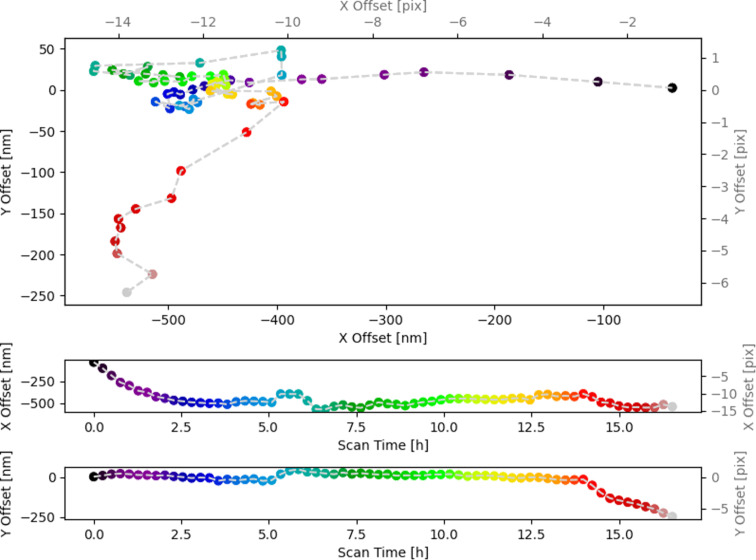
Drift offset **x**_tot_ tracked over time during the experiment (i.e., online) with drift correction enabled. The top graph shows the drift offset in *x* and *y* directions, with the bottom two graphs showing the individual dimensions on one axis and time on the other. The color coding of the data points in all three graphs indicates time.

**Figure 11:**
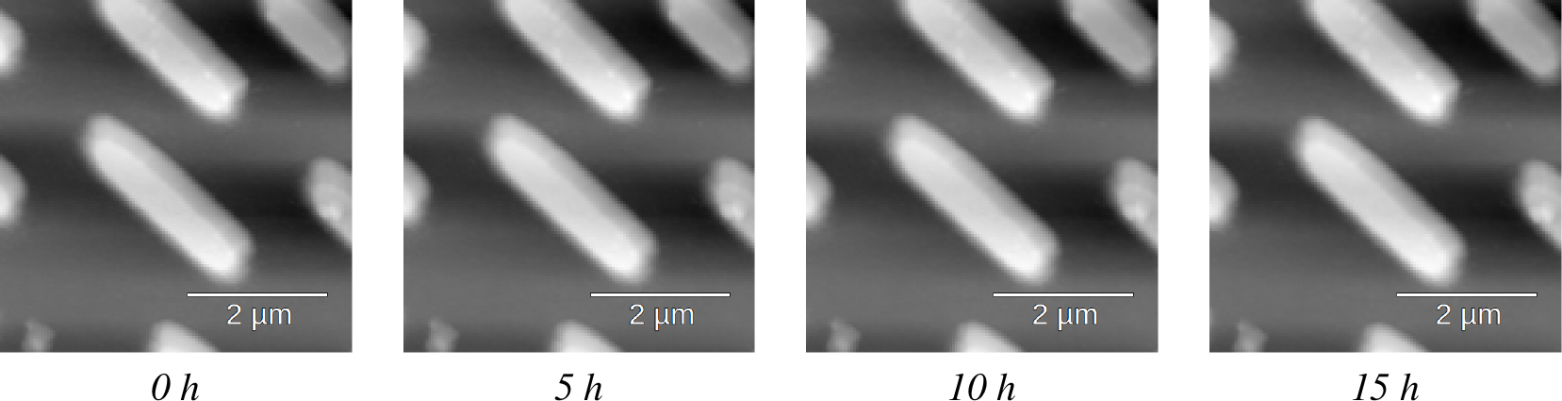
Sample scans collected over the duration of the experiment, at (a) 0 h, (b) 5 h, (c) 10 h, and (d) 15 h, respectively.

We decoupled our drift correction experiments into two phases, that is, scanning with our correction disabled and with it enabled. By scanning with our correction disabled, we were able to roughly characterize the thermal drift. This is useful in order to ensure our experiment configuration and reasonable drift correction parameters. For our test, we requested to scan a specific region over ∼2 h with the correction logic off. For a second experimental run, we again scanned a specific region over ∼16 h, this time with the correction logic on. To analyze the data, we visualized the measured drift offset **x**_tot_ for the case where correction was on in Figure 10. Here, we see that the tracked drift follows a non-linear trajectory, similar to the results in [14]. In contrast with that paper, we did not evaluate the ultimate precision and stability of our corrector; rather, we focused on validating that it tracked correctly and minimized drift between scans. A sample of images collected over the duration of the experiment can be found in Figure 11. The collected experimental data and configuration file used for this experiment can be found in Supporting Information File 3.

## Conclusion

afspm is a software framework for writing automated experiments in SPM, using generic commands and data structures and translating them for a microscope only as they are sent out to the microscope controller. afspm also has configuration file support to encourage reusable scripts, which decouples experiment-specifics from algorithmic logic. A mediation layer has additionally been developed, allowing for background tasks to intermittently take control of the microscope. This permits various “monitoring and fixing” automation tasks showcased in prior articles (e.g., tip functionalization) to be integrated in a straightforward manner. We demonstrated the ability to write and run generic code by testing the functionality on microscope controllers that expose each of the scripting interface types. We further validated its usage for running experiments via a drift correction experiment that successfully tracked drift over a reasonable time duration.

Some of afspm’s design decisions are worth highlighting. Its usage of a network-based IPC protocol and their definition in a configuration file allows experiments to be split up among multiple computers. This helps distribute computing resources, minimizing hardware constraints on experiments. In using a language-flexible data format and communication interface, users can use algorithms or programs that do not integrate with Python. We should highlight, however, that this flexibility is constrained to the communication protocol supported by our framework; framework features (such as configuration file parsing and some pre-existing software logic) will not be accessible.

We also highlight that the design decisions made here – encapsulating manufacturer-specific logic away from the main code, communicating between scripts over IPC interfaces, and configuration file parsing and instantiation via standalone processes – have broader appeal than just for SPM. Indeed, many experimental fields of study are hampered by a lack of standardization in their instruments’ communication interfaces, and may benefit from similar frameworks. We particularly highlight optical and electron microscopy as these instruments share common experimental workflows (e.g., searching a wider region to determine where to image) and may benefit from direct repurposing of automation logic developed for SPM.

In the future, our presented framework may benefit from integration with (or adaptation to) the Bluesky suite of Python packages [17]. That project’s focus on interoperability and coordination for multi-instrument experiments may prove useful as afspm progresses. Furthermore, the Bluesky data model may serve as a useful approach for organizing experimental data and metadata for later search and retrieval.

The development of automated tasks within a manufacturer-agnostic framework enables code sharing among the SPM community. This framework is a reasonable first step toward developing generic automation in SPM; as automation tasks are developed and integrated, it will make more experiments accessible to the community at large. We hope afspm encourages this collaboration and would be delighted to assist others in writing reusable automated scripts.

Our developed framework is available for free under an open source license from the Grutter Group Github and can be accessed at [24].

## Acknowledgements

N.S. thanks Erwan Leconte for his help in writing an initial version of the Omicron Research SXM controller and discussing nomenclature for parts of the framework, as well as Wyatt Behn and Natalie Greenberg for their assistance in reviewing drafts of the manuscript.

Icons included in the figures were used from the “Font Awesome 5 Free” icon set by FontAwesome (https://fontawesome.com/), contained in the fontawesome5 TeX package (https://ctan.org/pkg/fontawesome5), distributed under the terms of the SIL Open Font License (http://scripts.sil.org/OFL).

## Funding

The authors acknowledge NSERC of Canada, INTRIQ and RQMP via FRQNT of Quebec, and the National Research Council of Canada for funding. N.S. also acknowledges the McGill Engineering Doctoral Awards program and the RQMP Advanced Materials Academy for support.

## Supporting Information

File 1 Additional description of the framework.

File 2 Videos of the translator validation testing, performed on the various microscope controllers.

File 3 Data collected during experimental runs and the configuration file of these. The data folder contains the experimental scans as saved during the experiment. Config.toml contains the afspm configuration file as run. scan_metadata.csv holds the metadata file created by ScanMetadataWriter. drift_correction.csv contains the logged drift correction information created by the CSCorrectedScheduler (the legacy internal name for Drift Compensated Mediator). log.txt holds all logs sent out during the experiment.

## Data Availability

All data that supports the findings of this study is available in the published article and/or the supporting information of this article.
