# Supplementary material for: afspm: A framework for manufacturer-agnostic automation in scanning probe microscopy
Source: Beilstein J Nanotechnol. 2026 May 18;17:653–67. doi: 10.3762/bjnano.17.45 (PMC13202472; doi:10.3762/bjnano.17.45)
Supplement: File 1 — Additional description of the framework. [file Beilstein_J_Nanotechnol-17-653-s001.pdf]

BEILSTEIN JOURNAL OF NANOTECHNOLOGY

Supporting Information

for

afspm: A framework for manufacturer-agnostic automation in scanning probe microscopy

Nicholas J. Sullivan, Julio J. Valdés, Kirk H. Bevan and Peter Grutter

Beilstein J. Nanotechnol. **2026,** *17,* 653–667. doi:10.3762/bjnano.17.45

Additional description of the framework

License and Terms: This is a supporting information file under the terms of the Creative Commons Attribution License (https://creativecommons.org/licenses/by/4.0). Please note that the reuse, redistribution and reproduction in particular requires that the author(s) and source are credited and that individual graphics may be subject to special legal provisions.
The license is subject to the *Beilstein Journal of Nanotechnology* terms and conditions: (https://www.beilstein-journals.org/bjnano/terms)

Communication Protocol

Overview

In afspm, predefined commands and data structures are sent to and from network sockets in order to communicate between components (Figure S1). Here (and in the code), we use the term component to refer to a running algorithm that adheres to our communication protocol. In order to be transmitted across a medium, it is necessary to have a serialization method, that is, a process that translates the data into a sequence of bytes that may then be reconstructed on the other end. We chose Google Protocol Buffers [1] as our serialization library since it has multilanguage/multiplatform support, guarantees type safety, and avoids schema violations. To transmit our data between scripts we use ZeroMQ [2], a cross-language messaging library that simplifies the process of sending data between threads or processes. A number of network communication roadblocks are handled automatically, such as reconnection, network synchronization, and message queueing issues.

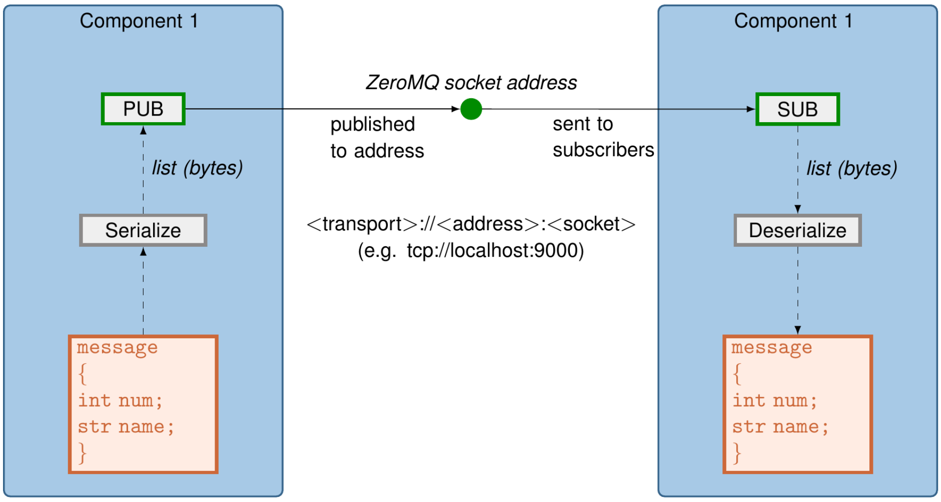

Figure S1: The communication protocol in afspm involves using Protocol Buffers to serialize data structures into a sequence of bytes (and the reverse deserialization process) and using ZeroMQ to send data between defined software components.

The wide number of languages supported and ability to run on all major platforms of both Protocol Buffers and ZeroMQ permits a component to be written in almost any programming language. Note that a component written in a different language will, in adhering to the communication protocol, only gain the ability to communicate with other components; the general functionality of the framework

will not be available, as it corresponds to code written in Python that can only be easily accessed by other Python scripts. Because of this, we suggest using Python where possible, restricting other language scripting to interfacing scripts (such as that used in Figure 4g of the main manuscript).

Our publisher–subscriber, request–response world

Communicating between the microscope and other components is decoupled in two paths: a publisher–subscriber path to receive events from the microscope and a control path to handle requests to the microscope (Figure S2). Any component that interacts with the microscope will likely subscribe to at least one message 'topic' from – and send at least one type of control request to – it. In this example diagram, a main Experiment script is subscribed only to `ScopeState` messages from the microscope and sends requests to start a scan, stop a scan, and change the `ScanParameters2d`. This script concerns itself only with deciding where to scan, and has some internal logic to determine these locations. A Visualizer script is subscribed only to `Scan2d` and `ScopeState` messages from the microscope, and does not send control requests. As we may expect, this script simply visualizes the latest scans to the user.

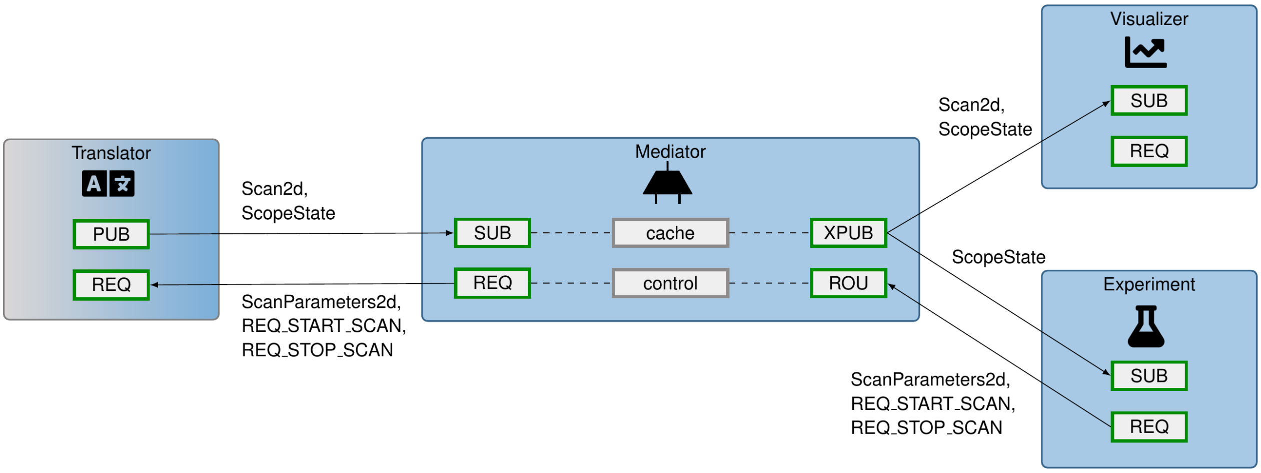

Figure S2: There are two main communication paths in afspm: a publisher–subscriber one, and a request–response one. The publisher–subscriber path allows the microscope to send information about its state, which all components can subscribe to. The request–response path allows components to take control of the microscope and request it perform actions or change its configuration. The mediator caches data in the publisher–subscriber path (to send out to new components) and mediates control of the microscope in the request–response path.

On the publisher–subscriber path, the microscope publishes different message types when changes have occurred. For example, if the scanning state of the microscope has changed, a new `ScopeState` message is sent; if a scan has finished, the new scan is read from disk into a `Scan2d` message and published. We have predefined most expected standard message types, but any user may define their own and compile it using the Proto Buffers utility. To better understand the publisher–subscriber data structures used and overall message format, sample structures are shown in Figure S3. Components need not receive all of the myriad message types the microscope may send – they may subscribe to the message types that interest them. These are differentiated in ZeroMQ via simple string keys denominated as ‘envelopes’, and the mapping from a given message type to a given envelope is configurable in afspm (with a reasonable default is provided).

```
message DataAspects {
  Size2u shape = 1;
  string units = 2;
}

message SpatialAspects {
  RotRect2d roi = 1;
  string length_units = 2;
  string angular_units = 3;
}

message ScanParameters2d {
  SpatialAspects spatial = 1;
  DataAspects data = 2;
}
```

(a) 2D Scan Parameters Schema

```
message Scan2d {
  ScanParameters2d params = 1;
  google.protobuf.Timestamp timestamp = 2;
  string channel = 3;
  string filename = 5;
  repeated double values = 4;
}
```

(b) 2D Scan Schema

Frame 1	`‘Scan2d’`	*Envelope*
Frame 2	`Scan2d Data`	*Serialized Data Structure*

(c) Publisher-Subscriber Message Format

Figure S3: Sample publisher–subscriber data structures and message format. (a) Spatial and data aspects both contain units defined for proper interpretation and manipulation (see Figure S4 for some of these geometric primitives). (b) The `params` and `channel` attributes indicate the data collected, the raw data is stored in `values`, and `timestamp` and `filename` distinguish it in time and on disk. (c) Messages published from the microscope are prepended an envelope that may be used to filter message types of interest. The default envelope creator uses the message name as the envelope string.

```
message Point2d {
  double x = 1;
  double y = 2;
}

message Size2u {
  uint32 x = 1;
  uint32 y = 2;
}
```

```
message Size2d {
  double x = 1;
  double y = 2
}

message RotRect2d {
  Point2d top_left = 1;
  Size2d size = 2;
  double angle = 3;
}
```

Figure S4: Sampling of geometric data structures used by our microscope data structures.

In the control path, components send requests to the microscope and receive responses accordingly. The microscope mediator functions as a router between the translator and all other components, ensuring that only one component is controlling the microscope at a given instance in time. Assuming a component is in control, it may send any of the defined control requests to the microscope; a component not in control may request control and add/remove experiment problems. By this mechanism, a component may temporarily pause regular running of the experiment (as described in the main manuscript).

When running an experiment, it is likely the automated components will at one time or another behave against one's expectations. To account for this, we defined a `ControlMode`, a 'switch' controllable during the experiment that may switch between `AUTOMATED` or `MANUAL` (with the former as default). Switching to `MANUAL` mode disengages any afspm components in control of the microscope and refuses any requests to gain control. When switched back to `AUTOMATED` mode, components can reconnect and continue the experiment.

For an overview of control path message types and the message format, see Figure S5.

```
enum ControlRequest {
  REQ_UNDEFINED = 0;
  REQ_ACTION = 1;
  REQ_ACTION_SUPPORT = 12;
  REQ_PARAM = 2;
  REQ_SET_SCAN_PARAMS = 3;
  REQ_SET_ZCTRL_PARAMS = 4;
  REQ_SET_PROBE_POS = 5;

  // Do not require control
  REQ_REQUEST_CTRL = 6;
  REQ_RELEASE_CTRL = 7;
  REQ_ADD_EXP_PRBLM = 8;
  REQ_RMV_EXP_PRBLM = 9;
  // Admin requests
  REQ_SET_CONTROL_MODE = 10;
  REQ_END_EXPERIMENT = 11;
}
```

(a) Control Request Schema

```
enum ControlResponse {
  REP_SUCCESS = 0;
  REP_FAILURE = 1;
  REP_CMD_NOT_SUPPORTED = 2;
  REP_NO_RESPONSE = 3;
  REP_ALREADY_UNDER_CONTROL = 4;
  REP_WRONG_CONTROL_MODE = 5;
  REP_NOT_IN_CONTROL = 6;
  REP_NOT_FREE = 7;
  REP_PARAM_NOT_SUPPORTED = 8;
  REP_PARAM_ERROR = 9;
  REP_WRONG_EXP_PROBLEM = 10;
  REP_ACTION_NOT_SUPPORTED = 11;
  REP_ACTION_ERROR = 12;
}
```

(b) Control Response Schema

```
enum ExperimentProblem {
  EP_NONE = 0;
  EP_TIP_SHAPE_CHANGED = 1;
  EP_DEVICE_MALFUNCTION = 2;
  EP_FEEDBACK_NON_OPTIMAL = 3;
  EP_THERMAL_DRIFT = 4;
}
```

(c) Experiment Problem Schema

```
enum ControlMode {
  CM_UNDEFINED = 0;
  CM_MANUAL = 1;
  CM_AUTOMATED = 2;
}
```

(d) Control Mode Schema

Frame 1	REQ_REQUEST_CTRL	*Command Request Enum*
Frame 2	EP_TIP_SHAPE_CHANGED	*Serialized Data Structure(s)*

(e) Control Request Message Format

Figure S5: Sample control data structures and message format. Panels (a) and (b) indicate enumerations for possible control requests and response. Panel (c) shows various experiment problems that may be flagged. Panel (d) indicates the mode the experiment may be in, with `AUTOMATED` being the default (where automation is running). Panel (e) shows a sample control request message, which consists of the control request enum and potentially one or more additional data structures, dependent on the request. In this case, a `REQ_REQUEST_CTRL` request is being sent, which demands an ExperimentProblem enum indicating the problem this requestor solves (if any). In this particular case, it is `EP_TIP_SHAPE_CHANGED`.

Caching and the Mediator

The mapping from message type to envelope is important for the mediator, which receives microscope events from the translator and republishes them. To permit newly instantiated components to understand the experiment state, the mediator caches prior events; when a new component connects to the mediator's publishing socket, all of the messages in its cache are sent out. The mediator maps message types to envelopes and stores them in a dictionary where the key is the envelope, with the exact logic being user-definable in the configuration file. As a simple example, one experiment may involve scanning a surface at two different 'granularities': a wide scan area resolution, to visualize the larger region, and a narrow-scan area resolution, to focus on particular adatoms of interest. Here, we would want to cache wide-area and narrow-area scans independently, so they both may be sent to components on startup.

Handling Units

All data-based structures have defined units; the various microscopes and developed automation components will likely differ in the units they function under. Handling these various units is thus important for reliable functioning of any experiment. We argue that each defined component should output data using its internally desired units and convert any received inputs into these same units. The responsibility for conversion thus lies with each individual component. Helper methods are provided to perform these conversions.

Spawning and Monitoring Components

The components to be run during an experiment can be defined in a single configuration file, and 'spawned' (or created) all-at-once or piecemeal via the command line. The term spawn here means that each component is instantiated as a child process, rather than a thread within a single process. In doing so, we ensure that faulty code in one component does not crash all components of the experiment, as each process has its own memory. Additionally, this allows for flexibility in

concurrent computing: As components are configured to communicate over TCP interfaces, they can be spawned on multiple computing devices.

The parent process that spawns components additionally monitors them, ensuring no frozen or crashed component is blocking the experiment. It accomplishes this by monitoring for 'heartbeats', explicit messages passed at a defined frequency by each component to the parent process. If a component has not sent a heartbeat within a defined timeframe, the monitor assumes it has frozen or crashed; it proceeds to force kill the process and respawn it.

Experiment Configuration

Our framework uses a TOML-based configuration file [3] to define components of an experiment, their settings, and the manners by which they communicate. Its general structure can be best understood from a sample configuration, shown in Figure S6. The file can be decoupled into three sections: general variables, intermediary classes, and components. General variables are the network sockets by which components communicate, as well as any additional variables that will be used to configure intermediary classes or components. In this example, they configure the control and publisher–subscriber paths between all the components. The components themselves are defined by having a boolean `component` attribute, with the `class` attribute used to determine how to load Python-based components. Note that during parsing of the file, string values are cross-referenced with keys to perform variable replacement (e.g., `'pub_url'` is replaced by `'tcp://127.0.0.1:9000'` in `translator_pub`). This allows variables to be defined in the config and used as a parameter for multiple components.

```
# --- URLs --- #
pub_url = "tcp://127.0.0.1:9000"
psc_url = "tcp://127.0.0.1:9001"

server_url = "tcp://127.0.0.1:6666"
router_url = "tcp://127.0.0.1:6667"

# --- Experiment Scan Aspects --- #
# - Physical Stuff - #
exp_physical_units = 'nm'
exp_origin = [0, 0]
exp_region = [200, 200]
exp_scan_size = [20, 20]

# - Data Stuff - #
exp_scan_res = [256, 256]
```

(a) General Variables

```
# ----- PubSub ----- #
[translator_pub]
class = 'afspm.io.pubsub.publisher.Publisher'
url = 'pub_url'

[scheduler_psc]
class = 'afspm.io.pubsub.cache.PubSubCache'
url = 'psc_url'
sub_url = 'pub_url'

[experiment_sub]
class = 'afspm.io.pubsub.subscriber.Subscriber'
sub_url = 'psc_url'

# ----- Control Logic ----- #
# [...]
```

(b) Intermediary Classes

```
[translator]
component = true
class = 'afspm.components.microscope.translators.gxsm.translator.GxsmTranslator'
publisher = 'translator_pub'
control_server = 'translator_server'

[scheduler]
component = true
class = 'afspm.components.microscope.scheduler.MicroscopeScheduler'
pubsubcache = 'controller_psc'
router = 'controller_router'

[experiment]
component = true
class = 'experiment.Experimenter'
subscriber = 'experiment_sub'
client = 'experiment_client'

scan_origin = 'exp_origin'
scan_region = 'exp_region'
scan_units = 'exp_scan_units'
subscan_size = 'exp_scan_size'
scan_res = 'exp_scan_res'
```

(c) Components

Figure S6: Excerpts from a sample configuration file, which can be divided into general variables defined at the beginning (a); intermediary classes (primarily input/output communication classes) (b); and components with their configuration (c).

Implementing Components in Other Languages

For a process to communicate with afspm components, it must be supported by Protocol Buffers and ZeroMQ, know the specific Protocol Buffers data structures employed by afspm, and know the ZeroMQ communication endpoints where it is expected to read/write data for a particular experiment. The first requirement will be either directly supported or there will hopefully be a third party library that allows these libraries to work. Knowledge of the data structures is straightforward: These are defined in afspm in 'proto' source files and can be compiled by the Protocol Buffers executable for the language of choice. The ZeroMQ communication endpoints are experiment-specific parameters traditionally defined in an afspm configuration file. Since our configuration file parser is written in Python and part of our framework, it is likely not accessible by the chosen programming language. As such, the implemented component will likely need to either implement its own configuration file format or maintain hardcoded endpoints in the script itself.

Note that the current version of afspm does not support spawning and monitoring components written in other programming languages. Subsequent versions may implement this support, if deemed worthwhile by the community.

Drift Correction

Algorithm overview

Using an image registration technique for drift correction, we estimate linear transformations M_{ij} between two scans S_i and S_j, where M_{ij} corresponds to a homogeneous linear transformation of the form $M = [R_{3\times2}|\boldsymbol{t}_{3\times1}]$ containing both a 3×2 rotation matrix R and a 3×1 translation vector $\boldsymbol{t}$. We track drift over time from the start of the experiment, considering a static sample coordinate system (SCS) and a dynamic tip coordinate system (TCS) that is changing throughout the experiment. We maintain a total mapping from TCS to SCS, denominated M_{tot}, which we initialize as $M_{tot} = [I_{3\times2}|\mathbf{0}_{3\times1}]$ (i.e., no difference between SCS and TCS). As the experiment runs, we grab time-subsequent scan pairs (S_i, S_j) and estimate the current drift $\widetilde{\Delta M}_{tot,k}$ between these, where k is a scan step. Simultaneously, we perform a first-order prediction $\widehat{\Delta M}_{tot,k}$ based on our prior drift estimates and use this to update our scan position when we begin a scan. Our drift correction logic, then, involves the following steps:

$$M'_{tot,k} = M_{tot,k-1} \cdot \widehat{\Delta M}_{tot,k}, \tag{S1}$$

$$M_{tot,k} = M'_{tot,k} \cdot \widetilde{\Delta M}_{tot,k}, \tag{S2}$$

where the drift predictive step occurs in Equation S1, used to set the scan region before performing a scan, and the estimation update occurs in Equation S2, performed once the scan finishes. By predicting drift at scan time and updating our predictions after the scan has finished, we attempt to reliably track drift throughout the experiment.

For estimating our drift mapping M_{ij} between two scans S_i and S_j, we use the feature detection approach taken in [4], where an algorithm detects common features in both scans and uses an outlier-robust mechanism to estimate the transformation necessary to align S_i and S_j spatially considering these feature pairs.

During the experiment, we keep track of the deviation between our drift estimates $\widetilde{\Delta M}_{tot,k}$ and drift

predictions $\widehat{\Delta M}_{tot,k}$. Deviation between these occurs when the drift vector has changed between scans. A large deviation tells us our tracking has failed and we may have lost a proportion of the region we aimed to track. Our configuration allows for the setting of a deviation threshold, above which we will force a rescan of the latest region with updated TCS coordinates. This rescanning aims to minimize loss of tracking due to such drift vector changes over the course of the experiment.

Usage in experiments

We chose to bundle our drift estimation and correction logic into microscope mediation logic, implementing an alternative mediator denominated a Drift Compensated Mediator (see Figure S7). Experiments can thus be designed considering a static coordinate system, as the data fed to connected components are in the drift-corrected sample coordinate system (SCS). For experiments where the time between scans permits significant changes in drift direction, an optional Drift Rescanner can be included; this script will perform rescans when the drift between scans exceeds some threshold.

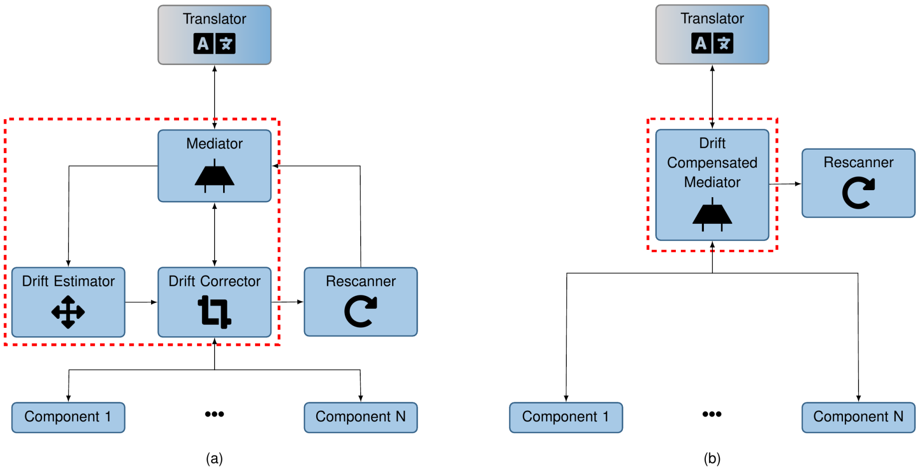

Figure S7: (a) Our drift correction logic involves drift estimation and correction such that experiments can be written in the drift-independent sample coordinate system (SCS), and requests sent to the microscope are converted to the true, drifting tip coordinate system (TCS). (b) We chose to bundle our drift correction logic with microscope mediation logic; drift correction can be enabled by swapping from the general Microscope Mediator to a Drift Compensated Mediator (the dashed red line indicates the logic encompassed by said mediator). An optional Rescanner can be enabled; it performs rescans when the difference between our drift estimates and predictions exceeds some threshold.

References

1. Protocol Buffers Documentation. https://protobuf.dev/ (accessed 2025-08-09).

2. ZeroMQ. https://zeromq.org/ (accessed 2025-08-09).

3. TOML: Tom's Obvious Minimal Language. https://toml.io/en/ (accessed 2025-08-09).

4. Diao, Z.; Ueda, K.; Hou, L.; Yamashita, H.; Custance, O.; Abe, M. *Appl. Phys. Lett.* **2023**, *122*, 121601. doi:10.1063/5.0139330.
